# Contrast-Induced Acute Kidney Injury: Definition, Epidemiology, and Outcome

**DOI:** 10.1155/2014/859328

**Published:** 2014-03-10

**Authors:** Felix G. Meinel, Carlo N. De Cecco, U. Joseph Schoepf, Richard Katzberg

**Affiliations:** ^1^Department of Radiology and Radiological Science, Medical University of South Carolina, Ashley River Tower, MSC 226, 25 Courtenay Drive, Charleston, SC 29425, USA; ^2^Institute for Clinical Radiology, Ludwig-Maximilians-University Hospital, 81377 Munich, Germany; ^3^Department of Radiological Sciences, Oncology and Pathology, University of Rome “Sapienza”—Polo Pontino, 04100 Latina, Italy; ^4^Division of Cardiology, Department of Medicine, Medical University of South Carolina, Charleston, SC 29425, USA

## Abstract

Contrast-induced acute kidney injury (CI-AKI) is commonly defined as a decline in kidney function occurring in a narrow time window after administration of iodinated contrast material. The incidence of AKI after contrast material administration greatly depends on the specific definition and cutoff values used. Although self-limiting in most cases, postcontrast AKI carries a risk of more permanent renal insufficiency, dialysis, and death. The risk of AKI from contrast material, in particular when administered intravenously for contrast-enhanced CT, has been exaggerated by older, noncontrolled studies due to background fluctuations in renal function. More recent evidence from controlled studies suggests that the risk is likely nonexistent in patients with normal renal function, but there may be a risk in patients with renal insufficiency. However, even in this patient population, the risk of CI-AKI is probably much smaller than traditionally assumed. Since volume expansion is the only preventive strategy with a convincing evidence base, liberal hydration should be encouraged to further minimize the risk. The benefits of the diagnostic information gained from contrast-enhanced examinations will still need to be balanced with the potential risk of CI-AKI for the individual patient and clinical scenario.

## 1. Introduction

Soon after modern iodinated contrast agents had been introduced, they have been causally linked to acute kidney injury (AKI) [1, 2]. A large number of noncontrolled observational studies have since investigated the frequency of AKI following intra-arterial and intravenous administration of contrast material (CM) and the overwhelming majority of studies found a significant risk [3]. Consequently, the risk of contrast-induced acute kidney injury (CI-AKI) has been widely accepted in medical literature and practice [4, 5]. Indeed, fear of CI-AKI is one of the most frequent reasons why CM is withheld from patients undergoing computed tomography (CT) and thus frequently compromises the diagnostic information gained from CT [6].

This traditional concept has been challenged recently, in particular for intravenous administration of contrast material [4]. This paradigm shift began when studies demonstrated a high rate of fluctuation in kidney function in patients without exposure to iodinated contrast material [7, 8], indicating that the existing observational studies on CI-AKI without a nonexposed control group are fundamentally flawed and likely to greatly overestimate the incidence of CI-AKI. Since then, a number of controlled studies have been performed examining the incidence of AKI in patients undergoing contrast-enhanced CT compared to patients undergoing noncontrast CT. A recent meta-analysis of 13 controlled studies found a similar or lower rate of AKI following contrast-enhanced CT compared to noncontrast CT indicating that CM is likely not the causative agent in the majority of postcontrast AKI cases [9]. It is, however, important to keep in mind that all of these controlled studies had a nonrandomized study design, which makes them vulnerable to selection bias, since patients perceived to be at risk for AKI are more likely to receive noncontrast CT examinations [2].

In view of this recent controversy, this paper reviews some fundamental aspects of the current concept of CI-AKI including its definition, epidemiology, and outcome and attempts to draw conclusions as to how the balance between avoiding AKI and gaining the necessary diagnostic information may need to be readjusted as a consequence of the recently published new evidence.

## 2. Definition

CI-AKI is commonly defined as a rise in blood urea nitrogen (BUN), serum creatinine, or a decline in estimated glomerular filtration rate (eGFR) occurring in a narrow time window—typically 24–72 hours—after administration of iodinated CM. In a recent meta-analysis of controlled studies on intravenous CM, definitions of AKI based on serum creatinine levels ranged from 0.3 to 0.5 mg/dL for an absolute increase and from 25 to 50% for a relative increase [9]. An absolute increase of serum creatinine by ≥0.5 mg/dL from baseline is a simple and still widely used definition of AKI [10]. Another commonly used definition is based on the criteria originally proposed by Barrett and Parfrey which consider CI-AKI to be present if an absolute increase in serum creatinine levels by ≥0.5 mg/dL or a relative increase in serum creatinine by ≥25% from baseline is observed within 72 hours after contrast exposure [11].

A more recent standardized definition of AKI was proposed by the Acute Kidney Injury Network (AKIN) and defines AKI if at least one of three conditions is met: (a) an absolute increase in serum creatinine levels by ≥0.3 mg/dL from baseline, (b) a relative increase in serum creatinine by ≥50% from baseline, or (c) a urine output reduced to ≤0.5 mL/kg/hour for at least 6 hours [12–14]. This definition is not designed for or specific to CI-AKI. However, the American College of Radiology has recommended using the AKIN criteria (occurring within 48 hours after the administration of iodinated CM) to define contrast-induced nephropathy in order to standardize the varying definitions found in the literature [15, 16]. Since urine output is not routinely measured in noncritically ill patients, the first two of the 3 criteria listed above have been used to define AKI in recent studies on CI-AKI with intravenous CM [15, 17].

The RIFLE classification is an alternative classification system which defines different stages of acute kidney injury (risk, injury, failure, loss, and end-stage renal disease) based on changes in serum creatinine or eGFR and urine output [12, 18]. In this classification, kidney injury is defined as a >100% increase in serum creatinine, a >50% decrease in eGFR, or a urine output of less than 0.5 mL/kg/h for 12 hours. This classification has been applied to postcontrast AKI [19, 20] but is not commonly used in this setting.

The incidence of AKI after contrast material administration greatly depends on the specific definition and cutoff values used to define AKI. For example, a prospective observational study of patients receiving intravenous contrast material for contrast-enhanced CT examined six different definitions of CI-AKI based on repeat serum creatinine measurements 48 to 72 hours after contrast exposure and the resulting incidence rates of CI-AKI ranged from 0–11%, depending on the definition used [21, 22]. Novel biomarkers of acute kidney injury such as cystatin-C, IL-18, KIM-1, and NGAL have been identified in recent years [23]. Further research is warranted to investigate whether these novel biomarkers may have a role in improving the risk stratification of patients receiving contrast material or in refining the diagnostic criteria of AKI.

Ultimately, all of these are arbitrary definitions of CI-AKI based on laboratory parameters. They are useful to create a numerical measure that can be statistically compared in clinical trials but bear no meaning for an individual patient. Only hard outcomes such as dialysis or death from renal failure are of real clinical significance.

Apart from these differences in the cutoff values defining AKI, differences in terminology merit consideration. Until a few years ago, any acute kidney injury occurring after administration to iodinated contrast material and not otherwise explained was assumed to be caused by contrast exposure and hence referred to as contrast-induced nephropathy (CIN) or contrast-induced acute kidney injury (CI-AKI) [2]. Considering the high rate of fluctuation in kidney function in patients without exposure to iodinated contrast material [7, 8], this terminology now appears greatly misleading, unless strictly reserved for the attributable excess rate of AKI caused by iodinated CM compared to a nonexposed control group. Considering that it is impossible to determine on an individual basis whether a decline in renal function was caused by CM, other predisposing factors, or a combination thereof, the term should be avoided in clinical practice when referring to individual patients. The terms “postcontrast AKI” or “postcontrast nephropathy” should be preferred since they do not infer a causal relationship. Furthermore, catheterization predisposes for renal compromise through mechanisms unrelated to contrast materials which are discussed in more detail below. Therefore, the terms “postcatheter nephropathy” or “catheter-induced nephropathy” should be used instead of “contrast-induced nephropathy” in patients after catheterization.

## 3. Epidemiology

### 3.1. Incidence of AKI in the Absence of CM Exposure

As discussed above, the epidemiology of CI-AKI has to be seen in the context of the background rate of fluctuations in kidney function and AKI observed in the absence of contrast exposure. Such fluctuations are particularly frequent in patients with chronic renal impairment and more frequent in hospitalized patients compared to outpatients due to a higher rate of comorbidities predisposing to AKI [7, 8]. In their meta-analysis of controlled studies of intravenous CM, McDonald et al. [9] report an overall AKI rate of 6.5% in the noncontrast CT group averaged over varying definitions of AKI. Davenport et al. found AKI incidence rates in the noncontrast group of 8.6% and 12.4% based on the AKIN and more traditional CIN criteria, respectively [17]. Using an absolute increase of serum creatinine 0.5 mg/dL from baseline to define AKI, McDonald et al. [10] found AKI rates in the noncontrast control group of 4, 9, and 13 percent for patients with a baseline creatinine of <1.5, 1.5–2, and ≥2 mg/dL, respectively. These background rates of AKI need to be taken into account when assessing whether CM exposure increases the frequency of AKI.

### 3.2. Incidence of AKI following Intra-Arterial Administration of CM

For a variety of reasons, the incidence of AKI is substantially higher following coronary angiographic procedures than following contrast-enhanced CT [22]. The patient population undergoing coronary procedures typically has more advanced vascular disease and a higher rate of hemodynamic compromise and is thus more predisposed for AKI than the average population undergoing contrast-enhanced CT [22, 24]. A larger volume of CM is typically applied in cardiac angiography, particularly if an intervention is performed during the procedure [22]. The site of CM injection (intra-arterial versus intravenous) may also have a direct influence possibly due to a higher initial concentration of CM in the renal vasculature, since it has been demonstrated for aortography that the risk of AKI is greater if the CM is injected immediately proximal to the renal arteries [4, 25]. Other authors, however, have challenged the notion of an increased AKI risk from intra-arterial compared to intravenous contrast administration [26, 27].

Furthermore, coronary angiography harbors a variety of iatrogenic risk factors for AKI, which may increase the risk of postcontrast AKI and are unrelated to the CM itself. It is well known that cholesterol emboli and microemboli from scraping of aortic plaque occur in a high percentage of patients during invasive angiographic procedures [28]. Iatrogenic (micro-) embolization of renal parenchyma may contribute to AKI following angiographic procedures [4]. Other potential complications of coronary angiography including arrhythmias, hemorrhage, myocardial infarction, or aortic dissection can all lead to hypotension or reduced cardiac output and thus contribute to postprocedural AKI which may be falsely attributed to the CM [8].

These factors need to be considered when interpreting the incidence rates of AKI after coronary angiography and related procedures. Reported incidence rates vary greatly depending on the patient population, the nature of the procedure, and definition of AKI. Postcontrast AKI is generally estimated to occur in approximately 5–15% of patients after PCI [29]. A decreased baseline renal function has consistently been found to be a strong predictor of postcontrast AKI risk [29]. The risk of post-PCI AKI has been shown to be significantly higher with high-osmolar CM compared to low-osmolar CM, but the iso-osmolar CM iodixanol has had conflicting results in further reducing risk even in vulnerable patients [29, 30]. Several studies have found evidence of a dose-dependent risk increasing with CM volume administered during the procedure [29, 31, 32]. Certain comorbidities (diabetes, proteinuria, hypertension, and dehydration) and nephrotoxic comedications further increase the risk of AKI following angiographic procedures [33, 34].

### 3.3. Incidence of AKI following Intravenous Administration of CM

The strongest currently available evidence on the incidence of CI-AKI following intravenous CM administration consists in a meta-analysis of controlled studies [9] and two additional large controlled studies [10, 17] which were published simultaneously and not included in the meta-analysis. The meta-analysis analyzed 13 controlled studies including more than 25,000 patients and found a similar or lower rate of AKI following contrast-enhanced CT compared to noncontrast CT [9]. This result was confirmed in subgroup analyses of patients with and without diabetes and with and without renal insufficiency. It was further independent of AKI definition and CM osmolality. The latter finding may not apply to high-osmolality contrast medium, since all but one study included in the meta-analysis used iso- and/or low-osmolality contrast media.

It is, however, important to keep in mind that all controlled studies included in the meta-analysis had a nonrandomized study design, which inevitably makes them vulnerable to selection bias, since patients perceived to be at risk for AKI are more likely to receive noncontrast CT examinations [2]. In the two more recent controlled studies on AKI after intravenous CM administration, sophisticated statistical methods of propensity score adjustment were used to neutralize differences in AKI risk factors between the contrast-enhanced and the noncontrast CT group and thus counteract the effects of selection bias. The results of these two studies have been partly conflicting. McDonald et al. found that after propensity matching there was no significant difference in AKI incidence between both groups [10]. Subgroup analysis was performed for patients with a baseline serum creatinine of <1.5, 1.5–2, and ≥2 mg/dL, and no significant difference between exposed and nonexposed patients was found in either group. The second study by Davenport et al. confirmed that there is no increased risk for patients with a baseline serum creatinine of <1.5 mg/dL [17]. This study, however, found a significantly increased risk for AKI after intravenous CM for patients with a baseline serum creatinine of >1.5 mg/dL, which further increased with higher baseline creatinine levels [17]. In a separate analysis of the same database, Davenport and colleagues analyzed the rates of AKI between patients undergoing contrast-enhanced and noncontrast CT using a risk group stratification based on eGFR instead of serum creatinine [15]. In this analysis, a significantly increased risk for the contrast exposed group was found in patients with an eGFR of <30 mL/min/1.73 m^2^ (OR 2.78, *P* = 0.02) and there was a trend of an increased risk in patients with an eGFR of 30–44 mL/min/1.73 m^2^ (OR 1.41, *P* = 0.07) [15].

How to explain and reconcile these conflicting results in patients with preexisting renal impairment is a subject of ongoing debate and has been discussed in detail elsewhere [2]. Reasons of this apparent discrepancy may include differences in patient population, contrast agent type, as well as differences in patient referral and nephropathy prophylaxis patterns [2]. Finally, propensity scoring can minimize the effect of selection bias but may not completely eliminate it, since the factors that prompted physicians to avoid a contrast-enhanced CT scan in individual patients may be more numerous and subtle than a retrospective analysis can account for. The ratio between contrast material dose and creatinine clearance has been suggested as a predictor of AKI risk from intravenous CM [35]. In hospitalized cancer patients, recent chemotherapy, hypertension, and treatment with bevacizumab have been identified as additional risk factors [36].

The best method to avoid selection bias would be to perform a randomized controlled trial randomizing patients to receiving noncontrast or contrast-enhanced CT. Such a trial has not been performed and will likely never be performed, since it appears unethical to randomize patients rather than use clinical judgment to carefully balance the potential diagnostic benefits and the risks of contrast administration (not limited to AKI) for the specific clinical indication and the risk factors of every individual patient.

Therefore, the true risk of CI-AKI from intravenous administration of CM is not and may never be precisely known. However, recently published evidence strongly suggests that the risk caused by CM is much smaller than previously thought based on noncontrolled studies. For patients with a baseline creatinine of <1.5 mg/dL and an eGFR of ≥45 mL/min/1.73 m^2^ the risk of CI-AKI is likely to be nonexistent. There is conflicting evidence for patients with impaired renal function; an increased risk of AKI from intravenous CM may exist in this patient population.

## 4. Outcome

Although self-limiting in most cases, postcontrast AKI carries a risk of more permanent renal insufficiency, dialysis, and death [29]. Levy and colleagues retrospectively compared the outcomes of 174 patients who developed AKI after CM administration for various procedures with matched controls who received CM but did not develop AKI [37]. This study found a significantly increased risk of in-hospital mortality (34% versus 7%) for those patients who developed post-AKI. However, it has been pointed out that AKI in most of these patients was probably not due to contrast material but other comorbid and iatrogenic risk factors [5, 38].

Most available evidence on the outcome of postcontrast AKI relates to intra-arterial CM administration for cardiac catheterization or other angiographic procedures. In a retrospective study of patients with preexisting renal insufficiency undergoing percutaneous coronary interventions (PCIs), Gruberg and colleagues found a significantly increased risk of in-hospital mortality (15% versus 5%) for those patients who had a ≥25% increase in serum creatinine within 48 hours following coronary procedures compared to those who did not [42]. A similarly striking increase in in-hospital mortality (21-22 versus 1–1.4%) in patients developing postcontrast AKI after PCI has been found in other studies [39, 40]. The incidence of AKI requiring dialysis after PCI is <1% in most published cohorts [29, 32, 39, 41]. In this small subgroup of patients, however, an even higher in-hospital mortality of 23–36% has been demonstrated [32, 39, 41, 42].

An adverse prognostic value of postcontrast AKI has also been demonstrated for longer-term mortality [38]. In patients without preexisting chronic kidney disease, 1 year cumulative mortality has been shown to be 8% and 3% in patients who did and did not develop postcontrast AKI after percutaneous coronary interventions, respectively [43]. In patients with preexisting renal disease, 1 year cumulative mortality increased from 7% to 23% if patients developed AKI after PCI. Similar results for the adverse effect of postcontrast AKI on 1 year mortality have been found in other study cohorts [39, 42]. One year mortality rates as high as 55% have been reported in patients who developed AKI requiring dialysis after PCI [41].

In summary, the literature has consistently demonstrated that patients who develop postcontrast AKI after catheterization procedures have a significantly higher risk of dying during the hospital stay and the next year. However, this finding must be interpreted with caution since it is derived from observational studies in which most postcontrast AKI patients had comorbidities that not only increase their risk of developing postcontrast AKI but also directly increase mortality. The association between postcontrast AKI and mortality, therefore, does not prove a causal relationship [38].

In contrast to intra-arterial CM for cardiac catheterization or other angiographic procedures, the risk of adverse outcome from postcontrast AKI is likely lower for intravenously administered CM. In an analysis of six prospective studies including >1,000 total patients undergoing contrast-enhanced CT with an overall postcontrast AKI rate of 5.1%, there was no case of dialysis or death resulting from postcontrast AKI [5].

## 5. Conclusions

In conclusion, the risk of AKI from CM, in particular when administered intravenously for contrast-enhanced CT, has been exaggerated by older, noncontrolled studies. More recent evidence from controlled studies suggests that the risk is likely nonexistent in patients with normal renal function, but there may be a risk in patients with renal insufficiency. However, even in this patient population, the risk of CI-AKI is probably much smaller than traditionally assumed. Since volume expansion is the only preventive strategy with a convincing evidence base [44], liberal hydration should be encouraged to minimize the risk for all patients, both with and without impaired renal function. Even though there is conflicting data, we believe it is still prudent to be more cautious in patients with significant renal impairment (a baseline creatinine of >2.0 mg/dL and/or an eGFR of <30 mL/min/1.73 m^2^). The benefits of diagnostic information gained from contrast-enhanced examinations will still need to be balanced with the potential risk of CI-AKI for the individual patient and clinical scenario.
